# Examining strain diversity and phylogeography in relation to an unusual epidemic pattern of respiratory syncytial virus (RSV) in a long-term refugee camp in Kenya

**DOI:** 10.1186/1471-2334-14-178

**Published:** 2014-04-01

**Authors:** Charles N Agoti, Lillian M Mayieka, James R Otieno, Jamal A Ahmed, Barry S Fields, Lilian W Waiboci, Raymond Nyoka, Rachel B Eidex, Nina Marano, Wagacha Burton, Joel M Montgomery, Robert F Breiman, D James Nokes

**Affiliations:** 1 Kenya Medical Research Institute (KEMRI)–Wellcome Trust Research Programme, Kilifi, Kenya; 2 United States Centers for Disease Control and Prevention, Nairobi, Kenya; 3 Emory University, Atlanta, USA; 4 Division of Global Health Protection, Center for Global Health, Centers for Disease Control and Prevention, Atlanta, USA; 5 School of Life Sciences and WIDER, Warwick University, Coventry, UK

**Keywords:** Attachment (G) protein, Displaced population, Epidemics, Genetic diversity, Genotype, Refugee, RSV

## Abstract

**Background:**

A recent longitudinal study in the Dadaab refugee camp near the Kenya-Somalia border identified unusual biannual respiratory syncytial virus (RSV) epidemics. We characterized the genetic variability of the associated RSV strains to determine if viral diversity contributed to this unusual epidemic pattern.

**Methods:**

For 336 RSV positive specimens identified from 2007 through 2011 through facility-based surveillance of respiratory illnesses in the camp, 324 (96.4%) were sub-typed by PCR methods, into 201 (62.0%) group A, 118 (36.4%) group B and 5 (1.5%) group A-B co-infections. Partial sequencing of the G gene (coding for the attachment protein) was completed for 290 (89.5%) specimens. These specimens were phylogenetically analyzed together with 1154 contemporaneous strains from 22 countries.

**Results:**

Of the 6 epidemic peaks recorded in the camp over the period, the first and last were predominantly made up of group B strains, while the 4 in between were largely composed of group A strains in a consecutive series of minor followed by major epidemics. The Dadaab group A strains belonged to either genotype GA2 (180, 98.9%) or GA5 (2, < 1%) while all group B strains (108, 100%) belonged to BA genotype. In sequential epidemics, strains within these genotypes appeared to be of two types: those continuing from the preceding epidemics and those newly introduced. Genotype diversity was similar in minor and major epidemics.

**Conclusion:**

RSV strain diversity in Dadaab was similar to contemporaneous diversity worldwide, suggested both between-epidemic persistence and new introductions, and was unrelated to the unusual epidemic pattern.

## Background

Displaced populations are reportedly at an increased risk of morbidity and mortality from acute respiratory infections [1,2], and viral respiratory pathogens contribute considerably to this disease burden [3]. Refugees often live in overcrowded settlements, suffer from malnutrition, can be highly mobile to their country of origin and into urban centres and frequently receive care from *ad hoc* and under-resourced health service providers [4]. In addition to the refugees themselves, refugee camps also host humanitarian aid workers from multiple organizations and many countries, contributing to uniquely diverse social contact patterns for refugees [5]. All of the above have a potential to modify pathogen diversity and transmission patterns in refugee camps. Given the consequent high risk for epidemics, it has been proposed that displaced populations should be given priority for respiratory infection prevention and control programs [3,6]. The origins, patterns, and diversity of disease-causing pathogens occurring in these populations are not well characterized; yet, greater understanding of these factors could have implications for the potential success of the control programs.

In 2007, to quantify the disease burden and understand the prevalence and seasonality of common respiratory viral pathogens [3], the Kenya Medical Research Institute (KEMRI), in collaboration with the United States Centers for Disease Control and Prevention (CDC), established respiratory illness surveillance in Kakuma and Dadaab refugee camps in Kenya [3]. Adenovirus and respiratory syncytial virus (RSV) were the 2 leading viral agents identified in patients diagnosed with either influenza-like illness (ILI) or severe acute respiratory infection (SARI) [3]. The surveillance in Dadaab camp revealed the occurrence of biannual peaks of RSV-associated illness in the camp [3] which was unusual in that while annual and biennial RSV cycles are observed elsewhere, we are unaware of any reports of twice yearly epidemics [7].

RSV clinical isolates can be classified into 2 genetically and antigenically distinct groups (A and B). These can be sub-classified into several genotypes within which further variation has also been documented [8]. Patterns of dominance in the prevalence of groups A and B have been observed to cycle in communities during consecutive epidemics (for example, A-A-B, A-A-B, etc..) and predominant genetic variants within the groups are usually replaced in successive epidemics [8-10]. These patterns have been hypothesized to reflect the interplay between the circulating RSV genetic or antigenic diversity and local factors, including herd immunity and social contact patterns [8]. Due to its notably higher variation compared with the rest of the RSV genome, and also being one of the known targets of host protective immunity [11,12], the gene coding for the RSV attachment (G) protein is frequently targeted in RSV molecular epidemiology studies [8].

We investigated the RSV group epidemiology and molecular diversity in the G gene of RSV positive specimens that were identified at the Dadaab refugee camp from September 2007 through November 2011. The detected virus strains were then compared by phylogeny with those identified globally during the same period (i.e. sequences from 22 different countries on 5 continents). The study aimed to (i) infer whether genetic diversity, at group and genotype level, played a role in the occurrence of the observed biannual epidemic cycles of RSV in Dadaab; (ii) determine and compare the degree of variation in the strains that occurred in the camp over time relative to those that were observed in stable populations; and (iii) determine the plausible origins of the strains that were circulating in the camp. Achieving these aims would increase our understanding on the mechanisms of RSV persistence in such populations.

## Methods

### Study population

The organization of the Dadaab refugee camp complex has been described elsewhere in detail [3]. The camp is located in Garissa County, North Eastern Kenya, about 100 km by road from the Somali border. While more than 95% of the county’s population is from Somalia, the refugee population is comprised of people from Somalia (62%), Sudan (23%), Ethiopia (3%), non-Kenyan east and central Africa (2.5%), and Kenya (9%). Within the Dadaab camp complex, sampling was undertaken in Hagadera (1 of 5 sites making up Dadaab complex). Patients with ILI were recruited from 1 of 4 health posts (outpatient clinics) while patients with SARI were recruited from the only camp hospital. All SARI patients were eligible for recruitment. The first 3 patients with ILI per day were also eligible for recruitment. Definitions of SARI and ILI, described previously [3], were as follows. ILI was defined as fever ≥38°C and cough or sore throat. SARI was defined as an admission to the hospital with the following age-specific criteria: (i) for an infant >1 week and < 2 months old, one or more of respiratory rate >60 per minute, severe chest indrawing, nasal flaring, grunting, fever ≥38°C, hypothermia < 35.5°C, or pulse oxygenation < 90%; (ii) for a child 2 months to < 5 years of age, cough or difficulty breathing with one or more of fast breathing for age (>50/min for a patient 2 months to < 1 year old or >40/min for children 1 to < 5 years old), chest indrawing or stridor in a calm child, inability to drink or breast feed, vomiting, convulsions, lethargy or unconsciousness, or pulse oxygen saturation < 90%; (iii) for a patient ≥5 years of age with fever ≥38°C, AND cough or sore throat, AND shortness of breath or difficulty breathing.

Specimen sampling involved the collection of a nasopharyngeal (NP) swab and an oropharyngeal (OP) swab, inserted into 1 mL of viral transport media (together in years 1 and 2, and then separately), and stored at 2-8°C for up to 96 h prior to shipment to the KEMRI-CDC laboratory in Nairobi [13]. Informed consent was obtained from all study participants or their guardians and the surveillance activities received approval from the Kenya National Ethical Review Committee and a non-research determination from CDC [3].

### Laboratory procedures

#### Samples analyzed

As previously described, the samples were initially screened for multiple respiratory viruses including influenza A and B, RSV, adenovirus, human parainfluenza viruses (1,2 and 3) and human metapneumovirus using singleplex real-time (reverse transcriptase [RT]) PCR assays [3,13]. The analysis reported here involved RSV sub-typing into groups A and B, and sequencing of G gene of the RSV-positive specimens. Only specimens with a cycle threshold (CT) value of 30 or lower were used as we had previously noted limited success with sequencing specimens that had CT values ≥30 for RSV (CNA personal communication). In the current work, we defined an “epidemic season” as a period during which ≥5 RSV cases were identified per month with no more than 1 month in which < 5 RSV cases were identified, and an epidemic as major if during the epidemic period >15 cases were identified during any month.

#### Viral RNA extraction and sub-typing

Viral RNA was extracted from the specimens using the QIAmp viral RNA extraction Kit (QIAGEN Ltd) with a starting sample volume of 140 μL and a final elution step with 60 μL of elution buffer. Sub-typing for RSV group A or B was processed through multiplex real-time RT-PCR, in a one-step fashion, with TaqMan probes that were specific for either RSV group A or B [14].

#### G gene amplification and sequencing

Extracted viral RNAs were reverse transcribed and amplified in a one-step reaction protocol (QIAGEN, Ltd) with primers targeting the entire RSV G gene and part of the F (fusion protein) gene (AG20 and F164) as previously described [15]. A microlitre of the resultant products was further amplified in nested PCR procedure with the primers BG10 and F1 [15]. Details of all primer sequences are presented in Additional file 1: Table A1. Success in amplification was confirmed on a 2% agarose gel (expected band size of ~830 bp) and the products purified using the GFX Illustra Kit (GE, Healthcare, UK Limited) and sequencing done using the BigDye 3.1 Chemistry on the 3130xl Sequencer (Applied Biosystems) with the nested PCR primers and additional group specific primers that ensured that all the target nucleotide positions were sequenced both in the forward and reverse directions [15]. Contigs were assembled to obtain the consensus in Sequencher 5.10 (Gene codes corporation, USA).

### Analysis of data

STATA 12 (Statacorp, Texas USA) was used for comparison of means (ttest) and proportions (prtest).

#### Phylogenetic analysis

RSV group A and B sequences were aligned separately in MAFFT software v6.884b [16]. Alignments were visually inspected and edited in Se-Al software v2.0 (http://tree.bio.ed.ac.uk/software/seal/). Phylogenetic analyses to classify the Dadaab sequences into genotypes and clades were carried out using MEGA 5 program [17] with trees being constructed using both Neighbor-joining and Maximum Likelihood methods. Confidence in branch clustering patterns was tested with 1000 bootstrap iterations. Phylogeographic analyses were carried out in BEAST 1.7.4 and chain convergence confirmed in Tracer v1.5 (http://tree.bio.ed.ac.uk/software/tracer/) (see Additional file 1: BEAST analysis). Trees were viewed within MEGA 5 or in Fig Tree program v.1.40 (http://tree.bio.ed.ac.uk/software/figtree/). Unique sequences are identified as single or groups of viruses which differ by at least one nucleotide in their sequenced G gene fragment from all other virus sequences in a specified location.

#### Definition of clades and sub-clades

The criteria for naming and assignment of sequences within the genotypes into further phylogenetically distinct categories (clades and sub-clades) were similar to that developed recently for the highly pathogenic avian influenza virus H5N1 [18]. The classification system provides information on the Dadaab strains with regard to the ancestor genotype from which they have diversified, for instance GA2.1 is a diversified form of GA2. Sequences qualified to be grouped into the same phylogenetic clade (on comparison of the G gene ectodomain) if they (i) occurred within the same branch supported by a bootstrap value of ≥60 (based on 1000 iterations on a neighbor-joining tree) and (ii) had an average genetic distance with other clades of >1.5% but an average genetic distance of less than 1.5% within the branch. This *ad hoc* clade naming system for Dadaab sequences was adopted in the absence of a currently existing consensus on naming RSV clades or new genotypes.

#### Comparison dataset

Two comparison datasets (for group A and group B) were compiled from the GenBank sequence database. These datasets consisted of all sequences from around the world that were collected from 2006 through 2011 and deemed potential co-circulating strains to those in Dadaab during the study period. The datasets were filtered to retain sequences whose length spanned the second hypervariable region of the RSV G and whose country of origin and date of collection could be ascertained (see Additional file 1 for information on the filtering process). The final comparison datasets included sequences from 22 countries in 5 continents (see Additional file 1: Table A2), including 3 sequences from Kilifi in coastal Kenya, 227 sequences from South Africa, and in total 649 sequences for group A and 505 sequences for group B collected from 2006 through 2011.

### GenBank accession numbers

The G gene sequences from the Dadaab strains reported in this study have been deposited into GenBank Database under the accession numbers KF156341 - KF156630.

## Results

A total of 336 RSV positive specimens collected at the Dadaab camp from September 2007 through November 2011 met the selection criterion for sub-typing and G gene sequencing. Of these, 99.7% were Somali, 80.4% were SARI patients, 56.3% were male, mean (median) age was 23.6 m (11 m), and mean (median) days of illness before presentation at health facility was 2.3d (3d). A comparison of the SARI versus (vs) ILI cases, respectively, showed that SARI cases had a higher proportion of males (57.9% vs 50.0%, P = 0.004) and were of a younger age (mean months = 17.2 vs 49.8, P ≤ 0.001; median months = 9 vs 24; under 2 years = 74.8% vs 42.4%, P ≤ 0.001). However, the number of days of illness before presentation at a health facility was comparable between SARI and ILI types (mean = 2.2d vs 2.4d, P = 0.670; median = 3d vs 3d).

**Figure 1:**
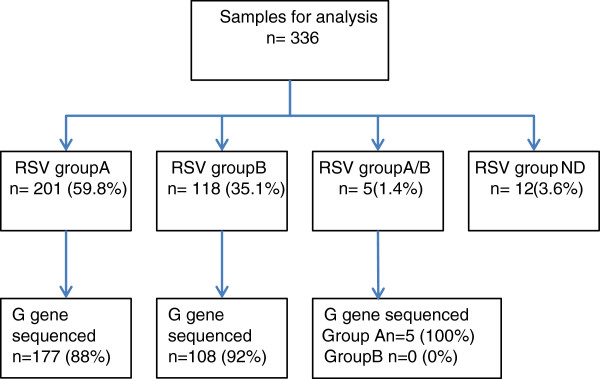
**Summary of results of sub-typing and sequencing of 336 RSV positive Dadaab samples that were selected from 2007–11 during the surveillance.** Note that of 5 co-infections of RSV group A and B, only group A sequences were obtained.

Among the 336 RSV positives, group A and B mono-infections were detected in 201 (59.8%) and 118 (35.1%) specimens, respectively, while 5 (1.4%) specimens appeared to be A-B co-infections. The remaining 12 (3.6%) were negative for both groups (Figure 1). Among the 201 group A infections 177 (88.1%) were successfully sequenced, while of the 118 group B infections 108 (91.5%) were successfully sequenced. All the 5 co-infections sequenced for group A G gene but none for group B; hence, in total there were 182 group A sequences (Table 1).

**Table 1:** Distribution of RSV specimens by group and epidemic (peak month), Dadaab, NE Kenya

**Peak Month**^**$**^	**RSV group A**^**§**^				**RSV group B**^**§**^			
	Identified (%)*	**Sequenced**	**Unique (%)**	**% GD**^**¶**^	**Identified**	**Sequenced**	**Unique (%)**	**%GD**^**¶**^
Jan 2008^#^	6 (14)	3	2 (67)	4.3	36	24	9 (38)	1.0
Jun 2008	34 (100)	22	10 (45)	5.9	0	-	-	-
Dec 2008^#^	66 (100)	66	12 (18)	0.6	0	-	-	-
Jun 2009	29 (94)	24	13 (54)	0.7	2	2	2 (100)	1.8
Dec 2009^#^	56 (100)	52	15 (29)	0.7	0	-	-	-
Mar 2011^#^	7 (8)	8	4 (50)	0.2	78	76	25 (33)	2.6
Inter-epidemic	8 (53)	7	6 (86)	2.7	7	6	5 (83)	2.4
Total^§^	206 (65.6)	182	62 (34.1)	2.4	123	108	41 (38.0)	2.6

^$^refers to the peak epidemic months of the epidemic periods we defined (see methods and Figure 2); January 2008 - span December 2007 to February 2008, June 2008 – span April 2008 to September 2008, December 2008 – span November 2008 to March 2009, June 2009 – span April 2009 to August 2009, December 2009 – span November 2009 to January 2010, Mar 2011 – span December 2010 to August 2011. Inter-epidemic period cases were causes observed outside the above defined epidemic periods and these occurred between March 2010 and October 2010, and October and November 2011.

^#^refers to the major epidemic peak month.

^§^The numbers in the respective column of RSV group A and group B include 5 co-infections, thus the number identified and in total are increased by 5 over numbers of mono-infections referred to in the text. Only Group A viruses were possible to sequence from co-infections hence numbers of Group A sequenced are also 5 more than for mono-infections alone.

*% Percentage of total group A and B identified that were group A.

^¶^refers to the average % genetic distance between the unique sequences which represent the % of nucleotide changes per every 100 nucleotides in the region between any the unique sequences.

### RSV group temporal patterns

**Figure 2:**
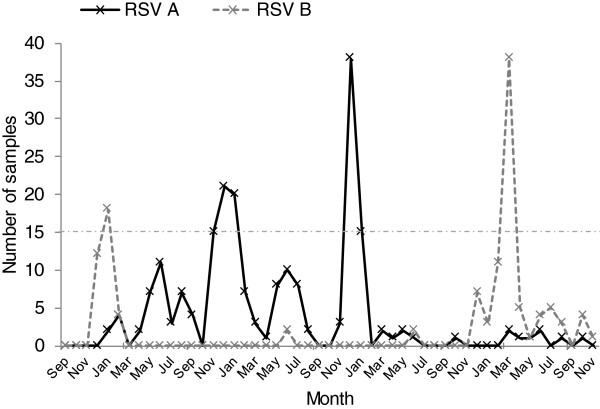
**Monthly cases of RSV group A and group B in Dadaab from September 2007 through November 2011.** RSV group A data points are joined by a black continuous line while group B data points are joined by a grey dashed line. The horizontal dot-dashed line marks 15 cases per month which was used to distinguish major from minor epidemics.

The 5 calendar years of surveillance at the Dadaab camp documented 6 RSV epidemic peaks, including 4 which were classified as major (Figure 2 and Table 1). The first epidemic involved predominantly group B strains and occurred from November 2007 through February 2008. This was followed by 4 consecutive epidemics (minor-major-minor-major) from April 2008 through February 2010 that were all predominantly associated with group A strains. Sporadic RSV positive cases were detected throughout 2010 but with no epidemic peak. Finally, a major epidemic associated principally with group B strains occurred after this series of group A epidemics starting in November 2010 and extending to August 2011 (Figure 2). Notably, during the 4 consecutive group A peaks, only 2 group B positive specimens were detected (both in mid- 2009) (Figure 2 and Table 1). Thus the group dominance pattern over the period was B-a-A-a-A-B (the lowercase letters represent minor epidemics).

### Genetic diversity and clustering of the Dadaab group A strains

**Figure 3:**
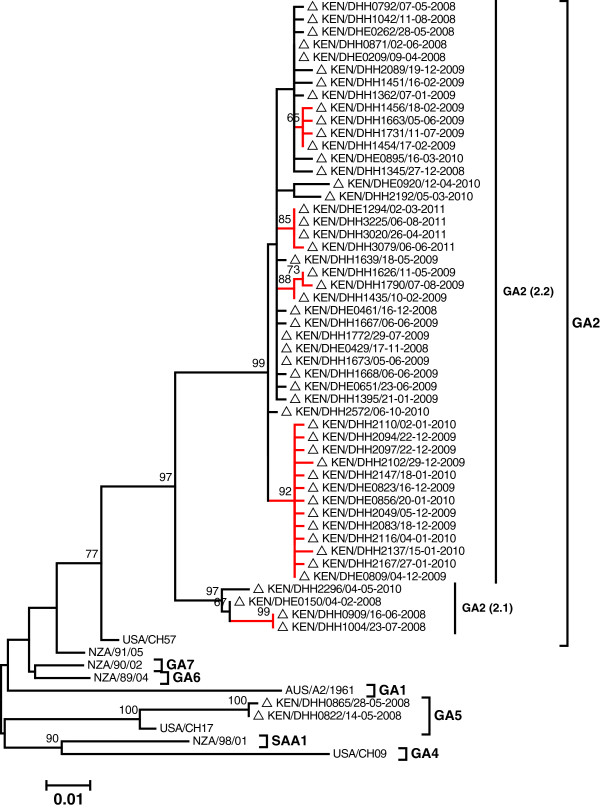
**A Maximum Likelihood tree showing the relatedness of the unique 52 RSV A Dadaab sequences.** Eight reference sequences within the previously identified RSV A genotypes (GA1-GA7 and SAA1) are included on the tree to allow determination of the genotypes of the Dadaab strain. Dadaab sequence taxon names are preceded by unfilled triangles. The tree was bootstrapped with 1000 iterations and only bootstrap support values ≥60 are shown. The clades identified within the GA2 genotype have been indicated on the tree as GA2 (2.1) and GA2 (2.2). The red branches identify those supported by a >60% bootstrap but did not meet the minimum genetic distance to be assigned into independent clades. Taxon names include country name/unique strain identifier/date of sampling.

The sequenced 182 Dadaab group A strains (including the 5 co-infections) showed high similarity over the 621 nucleotide region of overlap (nucleotides 301–912 on reference strain A2, accession number M11486). Throughout the study period only 28.6% (52/182) of these strains gave a unique sequence. A mean genetic distance of 1.2% was calculated for the whole Dadaab group A dataset, and 2.4% for the unique sequences alone. The number of unique sequences among the specimens sequenced for each epidemic peak ranged between 2 and 15 (median 10) and the proportion unique declined with number sequenced (Table 1). Within the previously described group A genotype classification (GA1-GA7, SAA1), 180 (98.9%) of the Dadaab group A strains fell into the GA2 genotype, with the 2 remaining stains (1.1%) falling into the GA5 genotype (Figure 3).

The Dadaab GA2 strains diversified into 2 main clusters (branches on the tree) that met our clade definition, and were named clade GA2 (2.1) and GA2 (2.2) (see Figure 3). The GA2 (2.2) clade is closely related to the NA1 (a GA2 sub-genotype) first reported in Niigata, Japan [19] and this clade included 96% (174/182) of the Dadaab group A strains. Within this GA2 (2.2) clade there were 4 sub-branches with a bootstrap support of >60% (coloured red on Figure 3), but these had limited between branch genetic distance (< 1.5%) and thus could not be assigned into further distinct clades. Nevertheless, these well-supported branches within GA2 (2.2) clade contained sequences mostly from a single epidemic (Figure 3 and Table 2), and thus probably represent a transmission cluster within a clade.

**Table 2:** Distribution of RSV genotypes and clades, by group and epidemic (peak month), Dadaab, NE Kenya

**Peak Month**^**$**^	**Jan 2008**	**Jun 2008**	**Dec 2008**	**Jun 2009**	**Dec 2009**	**Mar 2011**	**Inter-epidemic**^**♮**^
RSV group A							
GA2 (2.1)	2	3	0	0	0	0	1
GA2 (2.2)^♭^	1	17	66	24	52	8	6
*Sub-Branch 1*	*0*	*0*	*4*	*7*	*0*	*0*	*0*
*Sub-Branch 2*	*0*	*0*	*0*	*0*	*0*	*8*	*0*
*Sub-Branch 3*	*0*	*0*	*1*	*2*	*0*	*0*	*0*
*Sub-Branch 4*	*0*	*0*	*0*	*0*	*52*	*0*	*0*
GA5	0	2	0	0	0	0	0
RSV group B							
BA (2.1)	0	0	0	1	0	19	4
BA (2.2)	21	0	0	0	0	1	0
BA (2.3)	0	0	0	0	0	55	0
BA (2.4)	0	0	0	0	0	0	2
BA uncladed	3	0	0	1	0	1	0
Total	27	22	66	26	52	84	13

^$^The epidemic periods of the peaks are defined in Table 1 footnotes.

^♮^Inter-epidemic period refers to cases that were observed outside defined epidemic periods.

^♭^The sub-branches within GA2 (2.2) and the number of the sequences that fell under them are shown in italics in the corresponding row.

### Genetic diversity and clustering of the dadaab group B strains

Among the sequenced 108 Dadaab RSV group B strains, 41 gave a unique sequence over the 729-nucleotide long region of overlap in the G gene (nucleotide position 235 to 902 in reference strain CH18537, accession number M17213). All these group B Dadaab sequences were found to possess BA 60-nucleotide duplication and additionally showed the presence of a 6-nucleotide deletion within the first hypervariable region of the G gene. The mean genetic distance for the group B Dadaab dataset in total and for the unique sequences only was 2.2% and 2.6%, respectively. Similar to group A strains, the percentage of unique samples was negatively associated with sequence number (Table 1).

**Figure 4:**
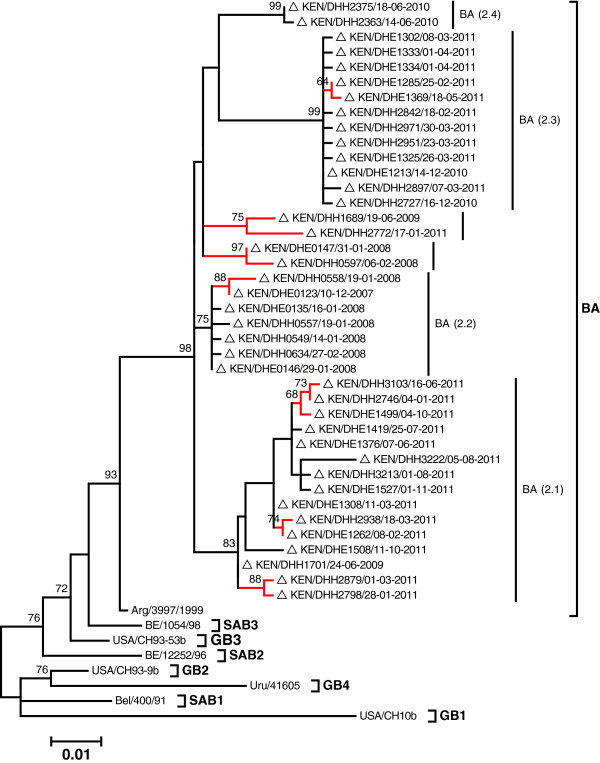
**A Maximum Likelihood tree showing the relatedness of the unique 41 RSV B Dadaab sequences.** Eight reference sequences representative of the 8 previously recognized RSV B genotypes (GB1-GB4, SAB1-3, and BA) are included on the tree to allow classification of the Dadaab strains into these genotypes. The Dadaab sequence taxon names are preceded by unfilled triangles. The tree was bootstrapped with 1000 iterations and only bootstrap support values ≥60 are shown. The clades identified within the BA genotype have been indicated on the tree (BA (2.1)-BA (2.4). The red branches identify those supported by a >60% bootstrap but did not meet the minimum genetic distance to be assigned into independent clades. Taxon name includes country name/unique strain identifier/date of sampling.

The phylogenetic relationship of the 41 unique Dadaab group B sequenced strains is shown in Figure 4. The analysis identified 6 branches with bootstrap support values >60% (indicated by vertical bars in Figure 4). However, of these only 4 had >1.5% genetic distance from other branches and were assigned into clades named BA (2.1) through BA (2.4) (see Figure 4). Of these, BA (2.1) clade showed most diversification (Figure 4) with a within clade mean genetic distance of 1.3% and 3 of its sub-branches had a bootstrap support of 60%. The Dadaab BA sequences did not cluster with any of the 11 previously described BA sub-genotypes (BAI-VI, BA7-10 and CB-B) [20-22] (see phylogeography RSV B section below).

The prevalence of the identified Dadaab group A and B genotypes and clades by epidemic peak is summarized in Table 2. Note that the sub-branch diversity for GA2 (2.2) clade was epidemic-specific whereas the diversity for BA 2.1 was within epidemic (Figures 3 and 4).

### Global phylogeography and diversity of RSV A strains during the period

**Figure 5:**
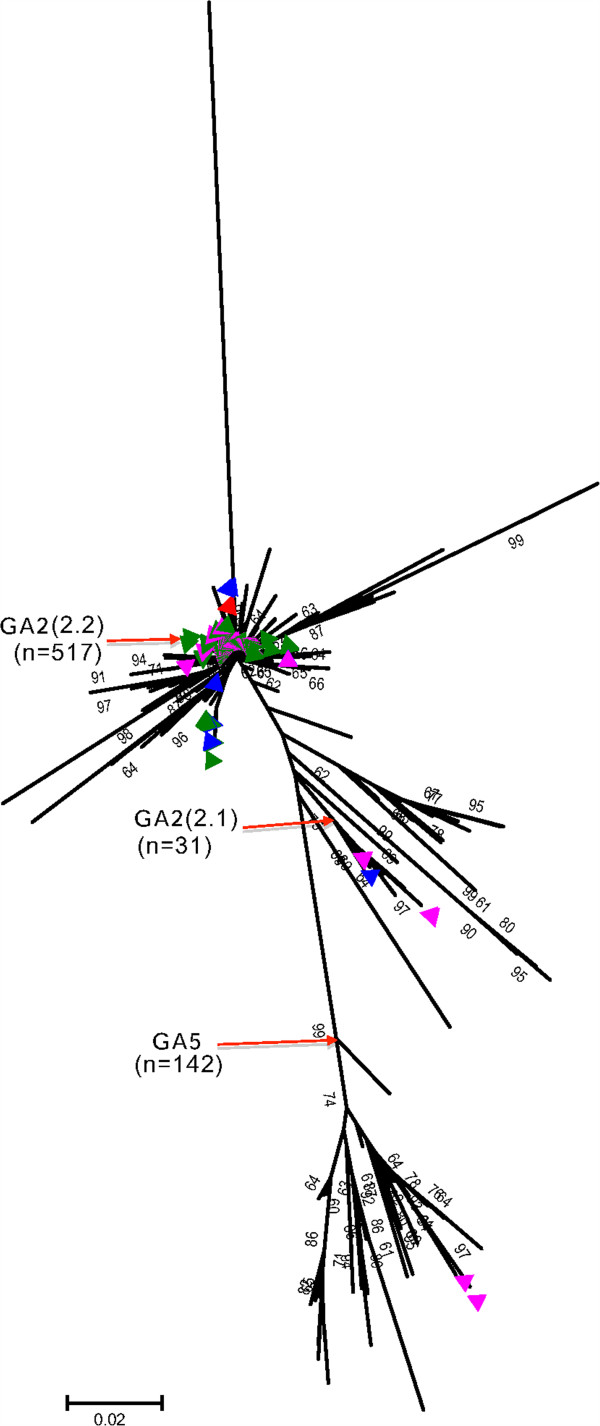
**A Neighbor-joining phylogenetic tree showing the relationships of all RSV A GenBank sequences (649) that we processed for the period 2006–11 combined with the Dadaab sequence data (182).** Sequences are compared in the second hyper-variable region of the G gene. The Dadaab sequences are indicated by the coloured triangles; their respective colours indicate the year of detection: maroon: 2007, pink: 2008, green: 2009, blue: 2010, red: 2011. The red arrows point to the nodes (or branches) that bring together sequences of the same genotype or clade identified at Dadaab and the number (n) indicates the number of sequences that fall within that branch. Tips without a triangle imply that the sequence was observed somewhere other than Dadaab. The tree is bootstrapped with 1000 iterations and whenever the percentage was greater than 60, the value is indicated next to the branch.

From the 649 RSV group A comparison sequences collected over the period 2006–11, we determined that globally 2 group A genotypes were circulating (GA2: 509, 78.4% and GA5: 140, 21.6%), and both were observed in Dadaab (Figure 5 and Table 3). A GA2 variant containing a 72 nucleotide duplication, the ON1 strain, was observed in Canada [23] and Malaysia [24] in 2011 that was absent from all other countries. Within the GA2, there was more diversity (~7 clades) than that observed at Dadaab (2 clades) (Figure 5). But consistent with the observations at Dadaab, the (2.2) clade (NA1) within the GA2 genotype was the most prevalent during this period globally (343/649, 52.9%) and had limited diversity. The Dadaab minority GA2 (2.1) clade strains represented 3.5% (23/649) in the global dataset and were observed in South Africa, Germany, Iran, and Netherlands. The 6 Dadaab GA2 (2.1) might have arrived in the camp from different sources because they occurred as 3 groups sandwiched between sequences of different countries (Additional file 2: Figure A1).

**Table 3:** Comparison of the global and Dadaab, Kenya datasets for RSV groups A and B

	**RSV group A**		**RSV group B**	
	**Global**	**Dadaab**	**Global**	**Dadaab**
Period (years)	2006-11	2007-11	2006-11	2007-11
Total Sequences^@^	649	182	504	108
Overall % Genetic distance	6.9	1.4	4.7	2.4
Sequences Unique (%)*	338 (52.1)	39 (21.4)	361(71.6)	24 (22.2)
Number of genotypes identified	2	2	4	1

^@^Sequences cover the the 2^nd^ hypervariable region of the G gene.

*The % refers to the sequences unique from the total sequences under the category.

The 140 GA5 sequences observed in the comparison dataset (versus 2 for Dadaab) were considerably diversified (see Figure 5) showing the presence of multiple well supported clades within (~11). The 2 Dadaab sequences fell into one of these clades that also contained sequences from Germany and Croatia. However, the Dadaab sequences still occurred on a sub-branch within the clade that was supported with bootstrap >70%, thus may not have been directly introduced from these countries (Additional file 3: Figure A2). Further information on the phylogenetic temporal clustering of Dadaab group A strains with the global dataset is described in a BEAST plot in Additional file 4: Figure A3.

### Global phylogeography and diversity of RSV B strains during the period

**Figure 6:**
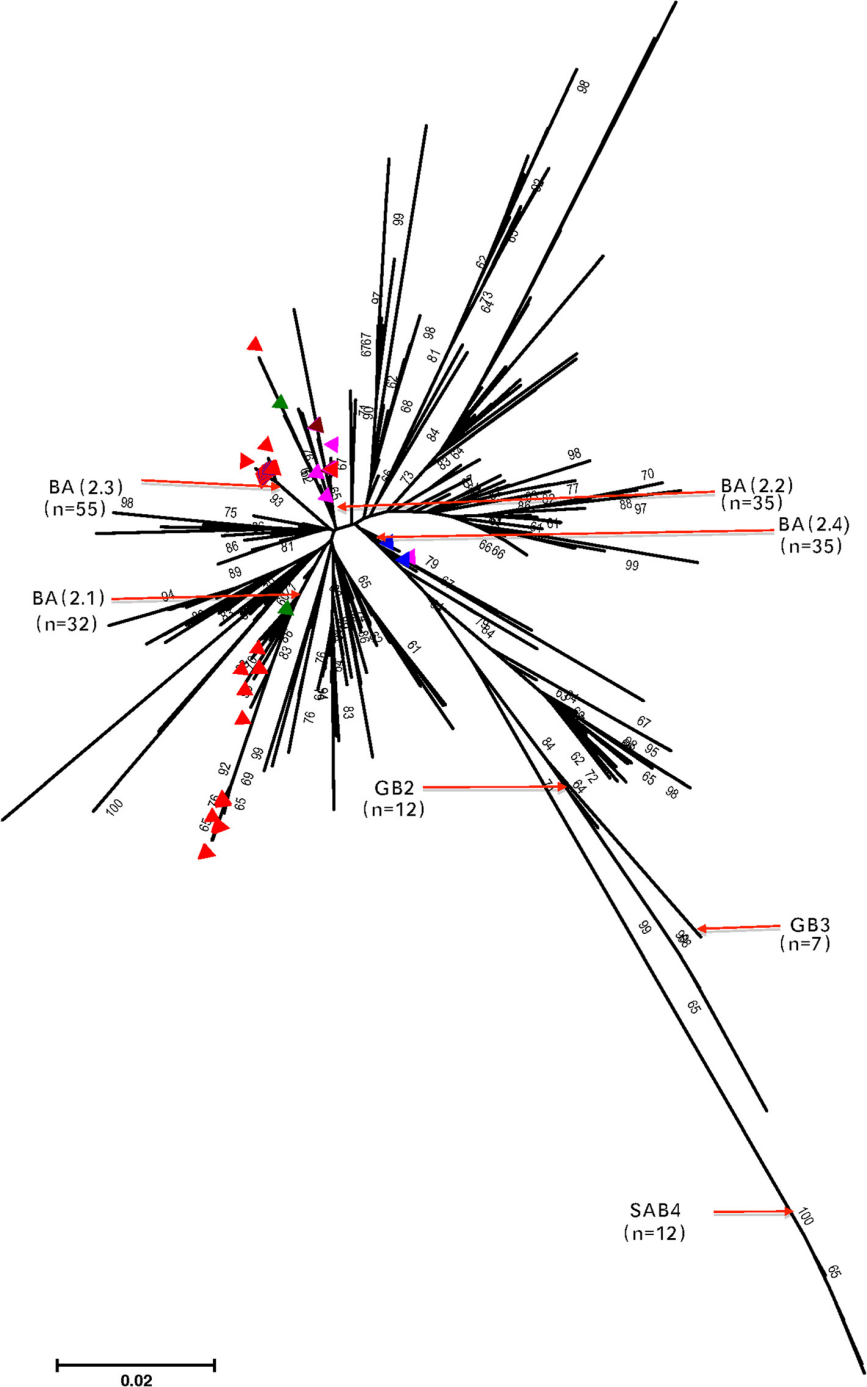
**A Neighbor-joining global phylogenetic tree showing the relationships of all the RSV B GenBank sequences (505) that we processed for the period 2006–11 and the Dadaab sequence data (108), i.e., a total of 613.** Sequences are compared in the second hyper-variable region of the G gene. The Dadaab sequences are indicated by the coloured triangles; their respective colours indicate the year of detection: maroon: 2007, pink: 2008, green: 2009, blue: 2010, red: 2011. The red arrows point to the nodes (or branches) that bring together sequences of the same genotype or clade identified at Dadaab and the number (n) indicates the number of sequences that fall within that branch. Tips without a triangle imply that the sequence was observed somewhere other than Dadaab. The tree was bootstrapped with 1000 iterations and whenever the percentage was greater than 60 the value is indicated next to the branch.

From the 505 RSV group B comparison dataset, 4 group B genotypes were observed during the period (BA: 474, 93.9%, GB2: 12, 2.4%, SAB4: 12, 2.4%, GB3: 7, 1.4%) but only the BA genotype was identified at Dadaab (Table 3). The phylogenetic relationships of all the Dadaab RSV B sequences together with the comparison dataset sequences are shown in Figure 6. Of the 4 defined Dadaab clades, only 2 were observed in other countries, namely BA (2.1) -- Latvia, Great Britain, and Iran, and for BA (2.2) – India, Kenya, and Korea. However, within BA during the period, several clades were circulating, most of which never occurred in Dadaab (Figure 6). Further information on the phylogenetic temporal clustering of Dadaab group B strains with the global dataset is described in a BEAST plot in Additional file 5: Figure A4.

## Discussion

This is the first report to describe RSV molecular epidemiology in a displaced population. The study characterizes the pattern of circulating RSV groups A and B genotypes, expanding the limited prior data for Kenya [25-27], and provides a phylogenetic analysis of the observed viral diversity in relation to worldwide circulating viruses. The study was motivated by the unusual biannual epidemic pattern in the Dadaab camp and whether this might be explained in the characteristics of the RSV genetic diversity.

Results show that of the 6 RSV epidemic peaks that occurred in Dadaab refugee camp from September 2007 through November 2011, 4 were predominated by group A consecutively, in a series of minor-major-minor-major, while the remaining 2, at either ends of the group A period, were predominated by RSV B. Though spanning only 5 calendar years, these observations are nevertheless unusual. Previous reports of biannual epidemics (e.g. Finland [28], Croatia [29], Switzerland [30], and Germany [31]) have involved alternating long and short inter-epidemic period, whereas in the present study, inter-epidemic periods were constant between the first 5 of the 6 peaks. Furthermore, the observation of 4 consecutive RSV A epidemics is at variance with previously reported time series [8-10,28] with pairs of epidemics of one group alternating between A and B. Nonetheless, our molecular analysis now extending to G variants within the group strains does support the previous observations by Mlinaric-Galinovic et al.*,* 2009 and 2012 in Croatia that virus diversity does not appear to be the driver of the unusual epidemic patterns [32,33].

Examining data from other geographical locations within Kenya [34] what is observed in Daadab could represent overlapping epidemiology from two regions with differing seasonal patterns. RSV epidemics peak at the Kenyan Coast and in the Western Kenya regions in Feb-March and in June-August, respectively. The minor and major peaks in Daadab seem to coincide with the peaks in the Western and Coastal regions, respectively. It would be of interest to look at the similarities of viruses from each location and epidemic to establish whether the unusual epidemic patterns in Daadab result from importation of viruses from other epidemiologically distinct regions in Kenya. Future work would also investigate if the RSV patterns in Dadaab can be explained by variation in seasonal forcing as has been shown for annual and biennial patterns analysed using a transmission dynamic model [10], or whether other factors (for example, birth rates and climatic factors) are necessary to account for biannual epidemics.

The G gene analysis showed that 3 RSV genotypes circulated in the Dadaab camp during our surveillance period (GA2: 62.1%, GA5: < 1% and BA: 37.2%) in qualitative agreement with the proportions observed in the GenBank comparison dataset (GA2: 44.1%, GA5: 12.1%, and BA: 41.1%). Other genotypes within RSV group B circulated elsewhere (GB2, GB3, SAB4) [35-37] but with considerably lower occurrence. The 4 consecutive RSV group A epidemics at Dadaab were almost exclusively GA2 genotype strains and were comprised of only 2 clades, GA2 (2.1) and GA2 (2.2), with the latter dominant (174/180, 96.6%). Although 7 clades occurred within the GA2 globally during this time frame, the GA2 (2.2) clade constituted the larger proportion of sequences within GA2 (343/509, 67.4%).

For both RSV group A and B, specimens collected at Dadaab during the same epidemic peak had limited diversity (Figures 3, 4 and Table 1). Overall, only 34.1% (62/182) of group A and 38.0% (41/108) of group B infections that were sequenced during the entire surveillance period gave unique sequences in the ectodomain G gene regions, and this further reduced to 21.4% (39/182) and 22.2% (24/108), respectively, when only the second hypervariable region of the G gene was considered (Table 3). This finding suggests that the frequency of new strain introductions into the camp may not have been high compared to stable populations, but that epidemics were associated with marked spread from small numbers of introduced or persisting virus strains. An alternative explanation is that the resolution offered by RSV sequencing of the G ectodomain alone was not adequate to distinguish closely related viruses that were arriving in the camp from different sources or at multiple times.

While Dadaab camp experienced a biannual epidemic pattern [3], in Kilifi District, located on the Indian Ocean Coast (500 km South), RSV epidemics were clearly annual [38]. Epidemics in Kilifi begin around November, coincident with the start of the major RSV epidemic season in Dadaab. Furthermore, over the study period the yearly group dominance pattern did not match for the two locations. For example, the major epidemic in Dadaab spanning the end of 2009 to early 2010 was almost entirely group A, whereas in Kilifi the epidemic of 2009–10 the two groups were co-dominant (Nokes et al., manuscript in preparation). These observations provide some evidence that RSV epidemics in Kenya are more regional than national as was observed for communities across distinct geographical locations in North America [39].

Notably, Dadaab camp did experience large in-migration during the surveillance period, in particular from 2006 through 2011, the camp population grew from less than 130,000 to almost 500,000 (JAA, personal communication) [4]. Given that the genetic variability of the virus in Dadaab was not greater than that observed globally, coupled with the observation that RSV group A strains were responsible for 4 epidemic peaks occurring sequentially, it is possible that higher rates of buildup of susceptible individuals or population density may have caused the unusual epidemiology observed. Investigations to elucidate the relative roles of genetic variability, waning immunity, and density of susceptible individuals within a population required for sustained recurrent epidemics of RSV will inform on mechanisms of RSV persistence.

This study is not without limitations. Firstly, the surveillance data described here were available over only 5 years, which is probably too short to display the underlying periodicities of RSV and its genetic variants. Secondly, not all eligible ILI and SARI patients were enrolled for screening, and only samples with a CT value of ≤ 30 were selected for sub-typing and sequencing. These could potentially allow under-detection of certain genotypes if their clinical manifestations were unique or if they had lower viral loads during peak infection. Thirdly, the phylogeography analyses, though representing one of the largest RSV datasets compared to date [40], are limited by the absence of comparison sequence data from throughout Kenya, the East Africa region and elsewhere on the continent. Furthermore, sequences from most other parts of the world include only a short fragment of the G gene and this reduces the phylogenetic signal when researchers are trying to resolve the origins of detected strains.

## Conclusion

In conclusion, we present the results of the first-ever study of RSV molecular epidemiology in a displaced population that also displayed an unusual epidemic pattern. We compare strains from these epidemics and with circulating genotypes within a large dataset compiled from 22 countries. Over a period spanning 5 calendar years, we observed that RSV genotypes circulating in the camp were similar to those identified to be co-circulating over the same period in stable populations but showed a reduced genetic variability within genotypes. Strain diversity did not seem to be the driving force behind observed unusual transmission patterns in the camp. Investigations are ongoing to assess whether the unusual epidemiologic patterns are associated with the changes in population size and in-migration.

## Competing interests

The authors declare that they have no competing interests.

## Authors’ contributions

Conceived and designed study: JAA, LWW, JMM, RFB, DJN; Generated field and laboratory diagnosis dataset: JAA, LWW, RN, NM, WB, RFB, BSF, RBE; Undertook sequencing LMM, JRO, CNA. Phylogenetic analysis and interpretation: CNA, LMM, JRO, LWW, BSF, DJN. Wrote manuscript: CNA, LMM, DJN, JAA, RFB, JMM, LWW. All authors read and approved the final manuscript.

## Pre-publication history

The pre-publication history for this paper can be accessed here:

http://www.biomedcentral.com/1471-2334/14/178/prepub

## Supplementary Material

Additional file 1 **Additional material.** Examining strain diversity and phylogeography in relation to an unusual epidemic pattern of respiratory syncytial virus (RSV) in a long-term refugee camp in Kenya. Click here for file

Additional file 2: Figure A1 Illustrates that within GA2 (2.1) where there is some diversity, Dadaab experienced probably 3 separate introductions for the 6 sequences seen (identified by triangle markers: pink, year 2008; blue, year 2010). Click here for file

Additional file 3: Figure A2 Illustrates the cluster within which Daadaab GA5 fell. The clusters contain sequences from Germany (DEU) and Croatia (HRV). The sequences cluster into 2 main groups with >70% boostrap support, illustrating that sequences might have not come directly from these countries and that these sequences are seen about one year earlier than those from Dadaab (pink triangles, year 2008). Click here for file

Additional file 4: Figure A3 A time-resolved maximum clade credibility BEAST tree showing the phylogenetic relationship of the unique RSV A sequences from Dadaab and the unique RSV A sequences from 16 other countries collected from 2006-2011. The taxa are coloured by the continent in which the sequences were sampled. The 16 countries included are Brazil (BRA), Canada (CAN), China (CHI), Croatia (HRV), Germany (DEU), Great Britain and Northern Ireland (GBR), Hong Kong (HKG), India (IND), Iran (IRN), Japan (JPN), Latvia (LVA), Malaysia (MYS), Netherlands (NLD), South Africa (ZAF), Thailand (THA), and South Korea (KOR). The clades identified in Dadaab are coloured in red. The taxon nomenclature includes three-letter abbreviation for the country of sampling/accession number/strain name. Click here for file

Additional file 5: Figure A4 A time resolved BEAST phylogenetic tree showing the relationship of the unique RSV B sequences from Dadaab and the unique RSV B sequences from 18 other countries collected from 2006-2011. The taxa are coloured by the continent the sequences were sampled. In addition to Kenya, the 18 other countries included are Brazil (BRA), China (CHI), Croatia (HRV), Great Britain and Northern Ireland (GBR), Hong Kong (HKG), India (IND), Iran (IRN), Japan (JPN), Latvia (LVA), Malaysia (MYS), Netherlands (NLD), South Africa (ZAF), Thailand (THA), Cambodia (KHM), Spain (ESP), Ireland (IRL), Vietnam (VNM), and South Korea (KOR). The Dadaab sequences are coloured in red, Kilifi in black. The taxon nomenclature includes three-letter abbreviation for the country of sampling/accession number/strain name. Click here for file
