# Supplementary material for: Examining strain diversity and phylogeography in relation to an unusual epidemic pattern of respiratory syncytial virus (RSV) in a long-term refugee camp in Kenya
Source: BMC Infect Dis. 2014 Apr 1;14:178. doi: 10.1186/1471-2334-14-178 (PMC4021307; doi:10.1186/1471-2334-14-178)
Supplement: Additional file 1 — Additional material. Examining strain diversity and phylogeography in relation to an unusual epidemic pattern of respiratory syncytial virus (RSV) in a long-term refugee camp in Kenya. [file 1471-2334-14-178-S1.docx]

**Additional file 1**

# Examining strain diversity and phylogeography in relation to an unusual epidemic pattern of respiratory syncytial virus (RSV) in a long-term refugee camp in Kenya

Agoti CN^1^, Mayieka LM^2^, Otieno JR^1^, Ahmed JA^2^, Fields BS^2^, Waiboci LW^2^, Nyoka R^2^, Eidex RB^2^, Marano N^2^, Burton W^2,4^, Montgomery JM^2,4^, Breiman RF^3,4^ and Nokes DJ^1,5^

*1. Kenya Medical Research Institute (KEMRI)–Wellcome Trust Collaborative Research Programme–Kilifi, Kenya*

*2. United States Centers for Disease Control and Prevention, Nairobi, Kenya*

*3. Emory University, Atlanta, USA*

*4. Division of Global Health Protection, Center for Global Health, Centers for Disease Control and Prevention, Atlanta, USA*

*5. School of Life Sciences and WIDER, Warwick University, UK*

**1. Comparison datasets*.***

Source of comparison dataset: Search online on <http://www.ncbi.nlm.nih.gov/pubmed>

Date of search: 26^th^ April 2013

Search term used (Empirically developed): ((((((((((((human respiratory syncytial virus G) NOT Kruppel-like) NOT truncated) NOT complete genome) NOT partial genome) NOT patent) NOT (N)) NOT bovine) NOT fusion) NOT F) NOT L) NOT ns1) NOT ns2].

Number of hits/sequences retrieved: 4510

These sequences were filtered to remain with sequences >270 nucleotides long, and for which the date of sampling (at least the year), and country of detection could be ascertained from the GenBank file.

Filtration methods: These involved our in-house biopython scripts.

The sequences were then aligned in MAFFT v6.884b and separated into RSV A and B. The alignments of the individual RSV groups were fine-tuned in Se-Al software and trimmed to remain with the 2^nd^ hyper variable region of the RSV G. Sequences that could not align with the rest were removed.

Nucleotide positions:

RSV A: Nucleotide positions 649-913 on reference strain A2, accession number M11486

RSV B: Nucleotide positions 649-973 on reference strain BA3833/99B, accession number AY333362

**2. Summary of the outcome of the stepwise filtering**

- Number of sequences started with: 4510
- Number of sequences dropped due to a sequence length <270 nucleotides: 631, remainder: 3879
- Number of sequences dropped due to absence of country information: 287, remainder: 3592
- Number of sequences dropped due to absence of date collection: 1818, remainder:: 1774
- Number of sequences dropped due to a collection date before 2006 or after 2011 dropped: 604, remainder: **1170.** These were then aligned in MAFFT and separated into RSV A and B as follows:
  - RSV A: 652
  - RSV B: 518

The separated group A and group B alignments were further adjusted in Se-Al and a few of the sequences that failed to align with the rest of the sequences were dropped. Ten sequences from Kilifi, Kenya identified as minority genomes within patients were also dropped but their 3 majority consensus representatives kept.

Our final comparison dataset constituted 649 group A and 505 group B sequences collected in 16 and 19 countries, for RSV A and B, respectively, from 2006-11. In the phylogeography analysis, these were merged with the Dadaab RSV A and B datasets (i.e. 182 RSV A and 108 RSV B) resulting into a total of 831 RSV A and 613 RSV B.

**Total dataset: 1444**

**3. Primer sequences**

**Additional file 1: Table S1.** Primers for RSV G gene amplification and sequencing

| Primer | Group | Gene | Position^1^ | Polarity | Function | Sequence (5’-3’) |
| --- | --- | --- | --- | --- | --- | --- |
| AG20 | A/B | G | 1-20 | + | PCR (outer) | GGGGCAAATGCAAACATGTCC |
| F164 | A/B | F | 164-187 | - | PCR (outer) | GTTATGACACTGGTATACCAACC |
| BG10 | A/B | G | 154-173 | + | PCR (inner) and sequencing | GCAATGATAATCTCAACCTC |
| F1 | A/B | F | 3-22 | - | PCR (inner) and sequencing | CAACTCCATTGTTATTTGCC |
| 523F | A | G | 538-557 | + | Sequencing | ATATGCAGCAACAATCCAAC |
| 523R | A | G | 557-538 | - | Sequencing | GTTGGATTGTTGCTGCATAT |
| 533F | B | G | 532-551 | + | Sequencing | TGTAGTATATGTGGCAACAA |
| 533R | B | G | 532-551 | - | Sequencing | TTGTTGCCACATATACTACA |

^1^ The positions of the primers for RSV A are given relative to the A2 strain (accession number: M11486) while the positions of RSV B specific primers are given relative to the reference strain CH18537 (accession number: M17213).

**4. Sampling periods of the datasets from the different countries**

A summary of this is given in supplementary table 1.

**Additional file 1: Table S2.** Sampling periods of the datasets from the countries included given as RSV A, B. Data derived from GenBank.

| **Year** | **2006** | **2007** | **2008** | **2009** | **2010** | **2011** | **Total** |
| --- | --- | --- | --- | --- | --- | --- | --- |
| Brazil (BRA) | 27, 7 | 47, 11 | 1, 1 | 15, 1 | 56, 16 | -, - | 146, 36 |
| Canada (CAN) | -, - | -, - | -, - | -, - | 2, - | 8, - | 10, - |
| China (CHN) | 13, - | 29, 1 | 7, 41 | 4, 6 | 7, 11 | 7, 1 | 67, 60 |
| Croatia (HRV) | -, 4 | 7, 1 | 6, 3 | -, - | -, - | -, - | 13, 8 |
| Germany (DEU) | 25, - | 48, - | 2, - | -, - | -, - | -, - | 75, - |
| Great Britain and N Ireland (GBR) | -, 1 | 4, 3 | 5, 5 | 11, 4 | 4, 6 | -, - | 24, 19 |
| Hong Kong (HKG) | 4, 2 | 4, 1 | 5, 2 | 1, 5 | 5, 9 | 13, - | 32, 19 |
| India (IND) | -, 7 | -, 1 | -, - | 5, - | 2, - | -, - | 7, 8 |
| Iran (IRN) | -, - | 2, - | 4, - | -, 8 | -, - | -, - | 6, 8 |
| Japan (JPN) | 1, 10 | 16, 32 | 6, 8 | 6, 9 | 28, 13 | -, - | 57, 72 |
| Latvia (LVA) | -, - | -, - | -, - | 1, 1 | 9, 2 | -, - | 10, 3 |
| Malaysia (MYS) | -, 1 | -, 1 | -, - | -, 31 | -, - | 1, - | 1, 34 |
| Netherlands (NLD) | -, 0 | 3, 1 | 9, 3 | -, - | -, - | -, - | 12, 4 |
| South Africa (ZAF) | 46, 31 | 74, 10 | 29, 5 | 4, 28 | -, - | -, - | 153, 74 |
| S. Korea (KOR) | -, - | -, - | 5, 10 | 11, 9 | 8, 5 | -, - | 24, 24 |
| Thailand (THA) | 12, 7 | -, 0 | -, 12 | -, - | -, - | -, - | 12, 19 |
| Cambodia (KHM) | -, - | -, 1 | -, 12 | -, 47 | -, - | -, - | -, 60 |
| Spain (ESP) | -, 3 | -, 10 | -, 5 | -, - | -, - | -, - | -, 18 |
| Ireland (IRL) | -, 5 | -, 6 | -, 0 | -, 4 | -, - | -, - | -, 15 |
| Vietnam (VNM) | -, - | -, - | -, - | -, - | -, 18 | -, 1 | -, 19 |
| Kenya^♯^(KEN) | -, - | -, 7 | 61, 20 | 92, 3 | 21, 9 | 8, 73 | 182, 111 |
| Total | 128, 78 | 234, 86 | 140, 127 | 150, 155 | 143, 89 | 37, 76 | 649, 611 |

**^♯^** Sequences from Kenya refer to sequences reported in this study except for 3 sampled at Kilifi that are present in Gen Bank (1 in 2007 and 2 in 2008). Kenya (Dadaab) data are currently deposited into GeNBank.

A dash is indicated if the data for a country for either group were absent in our final filtered dataset.

**Total number of countries = 22**

5. **BEAST Analysis**

Samples that did not have a full date of collection were given an estimated date as follows: if only the day of collection was missing, then the 15^th^ day of the month was assumed to be the date of sample collection. If both the day and the month were lacking, then 31^st^ June for that year was assumed to be the date of sample collection.

Prepared unique datasets for each country for comparison with the Dadaab dataset

- RSV A: 400 sequences (361 Genbank sequences compared with 39 Dadaab sequences
- RSV B: 390 sequences (366 Genbank sequences compared with 24 Dadaab sequences)

Length of chains: 50 million, sampling after every 2500 generations
