# Supplementary figures and images for: Examining strain diversity and phylogeography in relation to an unusual epidemic pattern of respiratory syncytial virus (RSV) in a long-term refugee camp in Kenya
Source: BMC Infect Dis. 2014 Apr 1;14:178. doi: 10.1186/1471-2334-14-178 (PMC4021307; doi:10.1186/1471-2334-14-178)

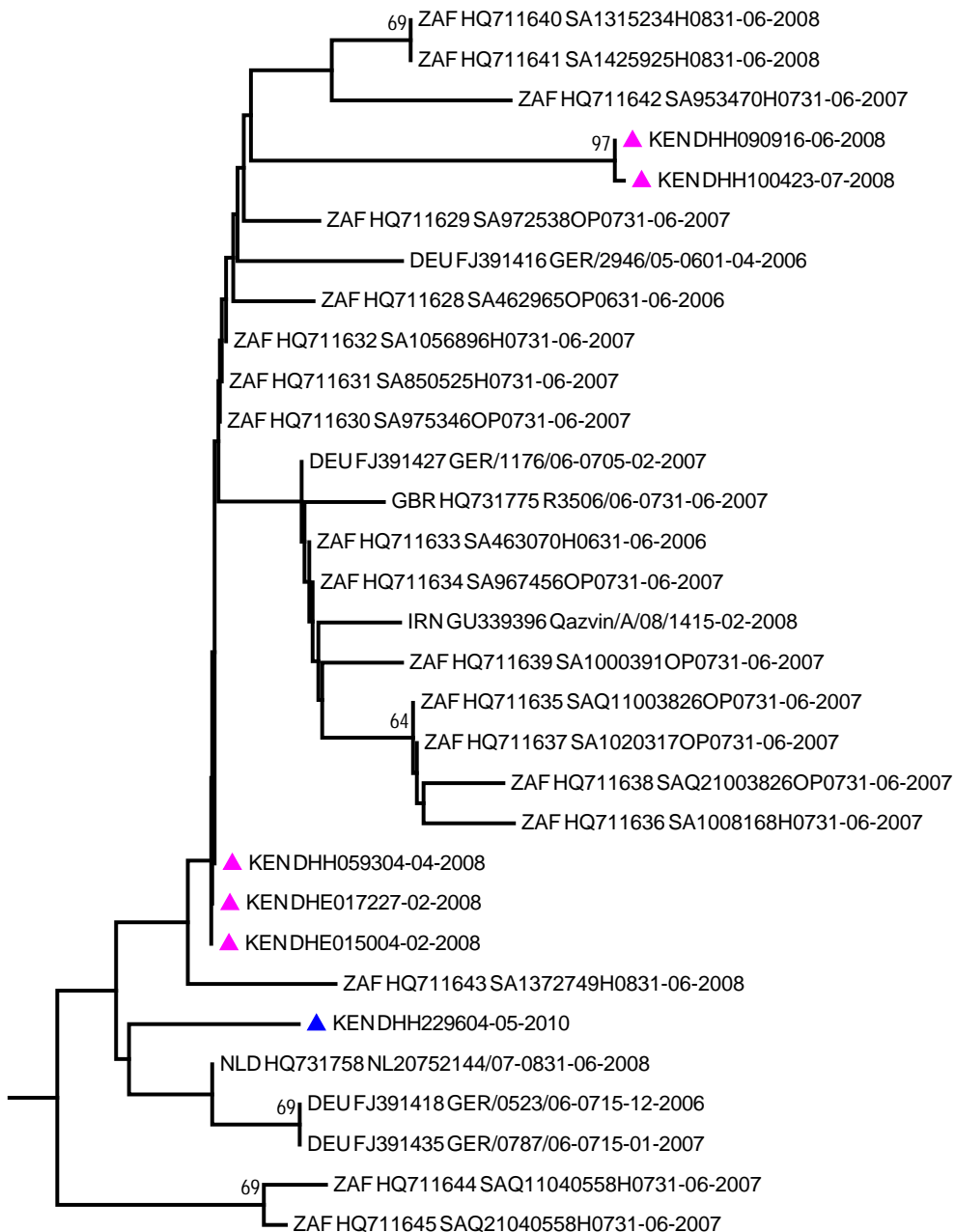

0.002

Supplement: Additional file 2: Figure A1 — Illustrates that within GA2 (2.1) where there is some diversity, Dadaab experienced probably 3 separate introductions for the 6 sequences seen (identified by triangle markers: pink, year 2008; blue, year 2010). [file 1471-2334-14-178-S2.pdf]

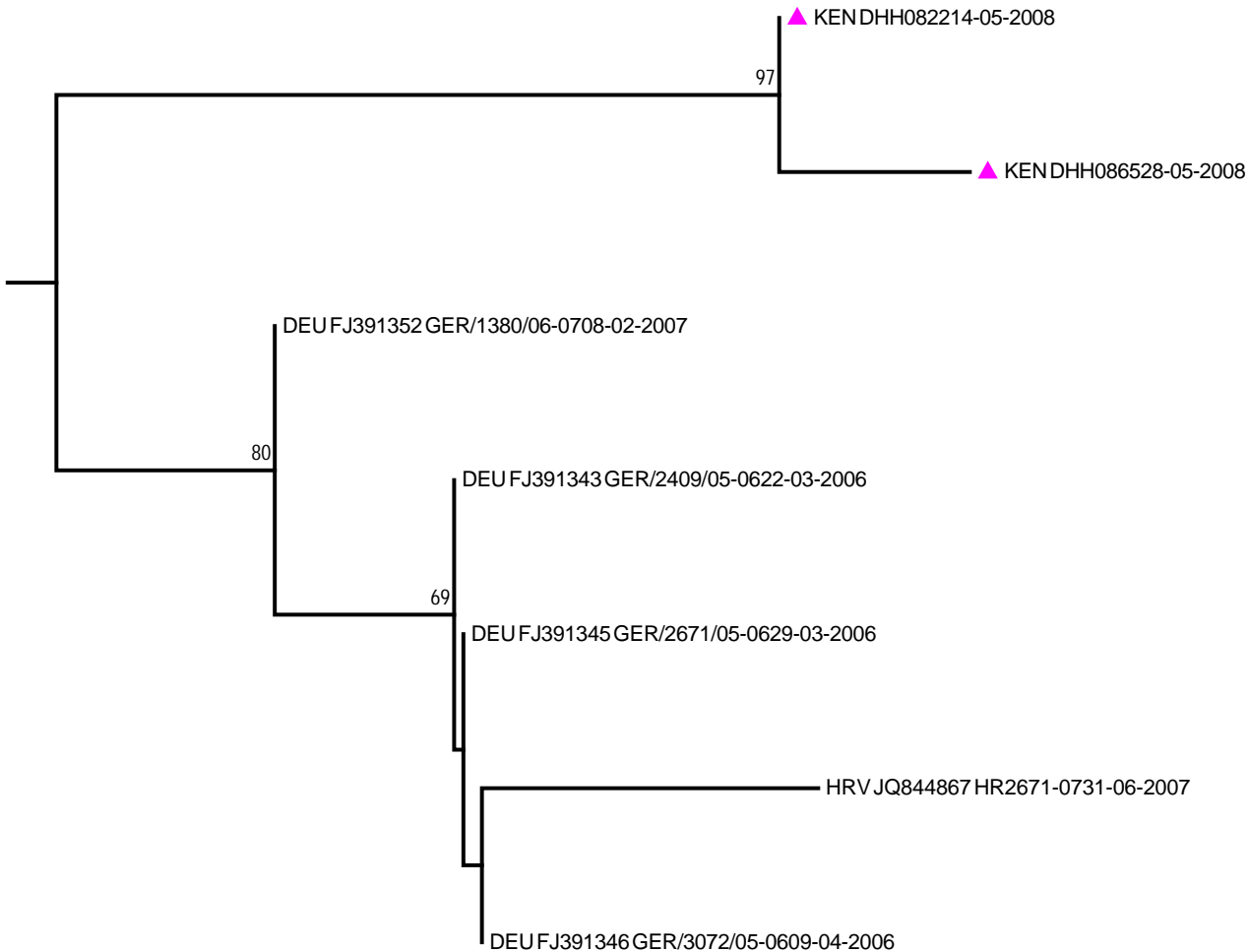

0.005

Supplement: Additional file 3: Figure A2 — Illustrates the cluster within which Daadaab GA5 fell. The clusters contain sequences from Germany (DEU) and Croatia (HRV). The sequences cluster into 2 main groups with >70% boostrap support, illustrating that sequences might have not come directly from these countries and that these sequences are seen about one year earlier than those from Dadaab (pink triangles, year 2008). [file 1471-2334-14-178-S3.pdf]

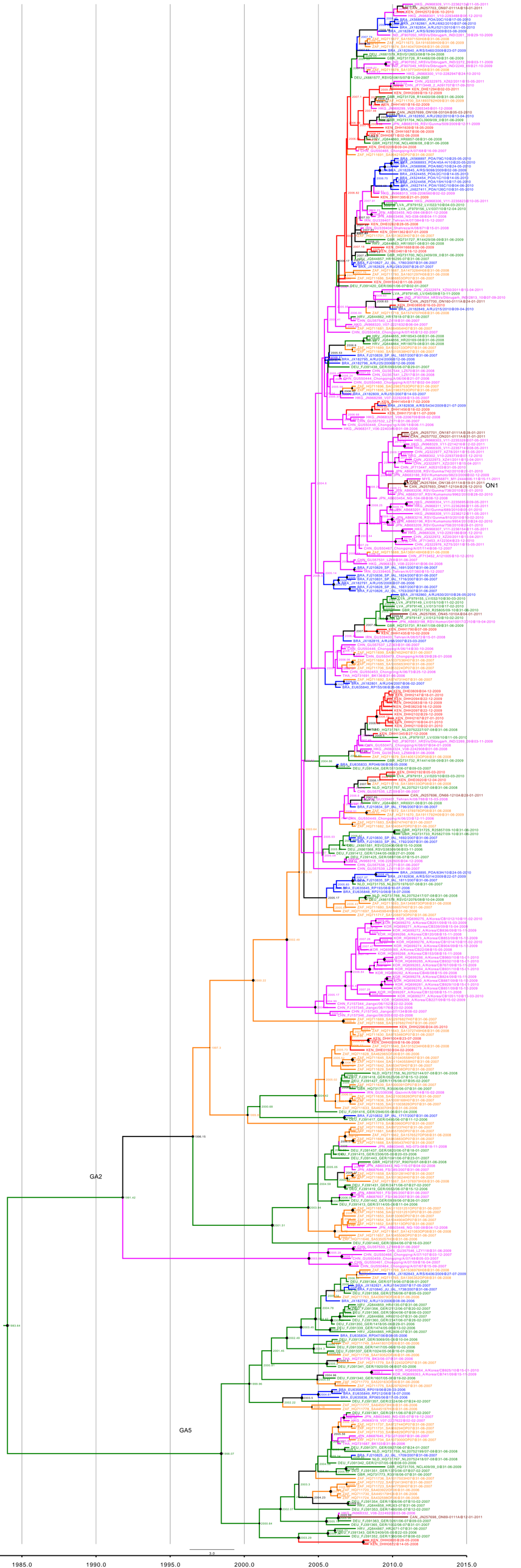

Supplement: Additional file 4: Figure A3 — A time-resolved maximum clade credibility BEAST tree showing the phylogenetic relationship of the unique RSV A sequences from Dadaab and the unique RSV A sequences from 16 other countries collected from 2006-2011. The taxa are coloured by the continent in which the sequences were sampled. The 16 countries included are Brazil (BRA), Canada (CAN), China (CHI), Croatia (HRV), Germany (DEU), Great Britain and Northern Ireland (GBR), Hong Kong (HKG), India (IND), Iran (IRN), Japan (JPN), Latvia (LVA), Malaysia (MYS), Netherlands (NLD), South Africa (ZAF), Thailand (THA), and South Korea (KOR). The clades identified in Dadaab are coloured in red. The taxon nomenclature includes three-letter abbreviation for the country of sampling/accession number/strain name. [file 1471-2334-14-178-S4.pdf]

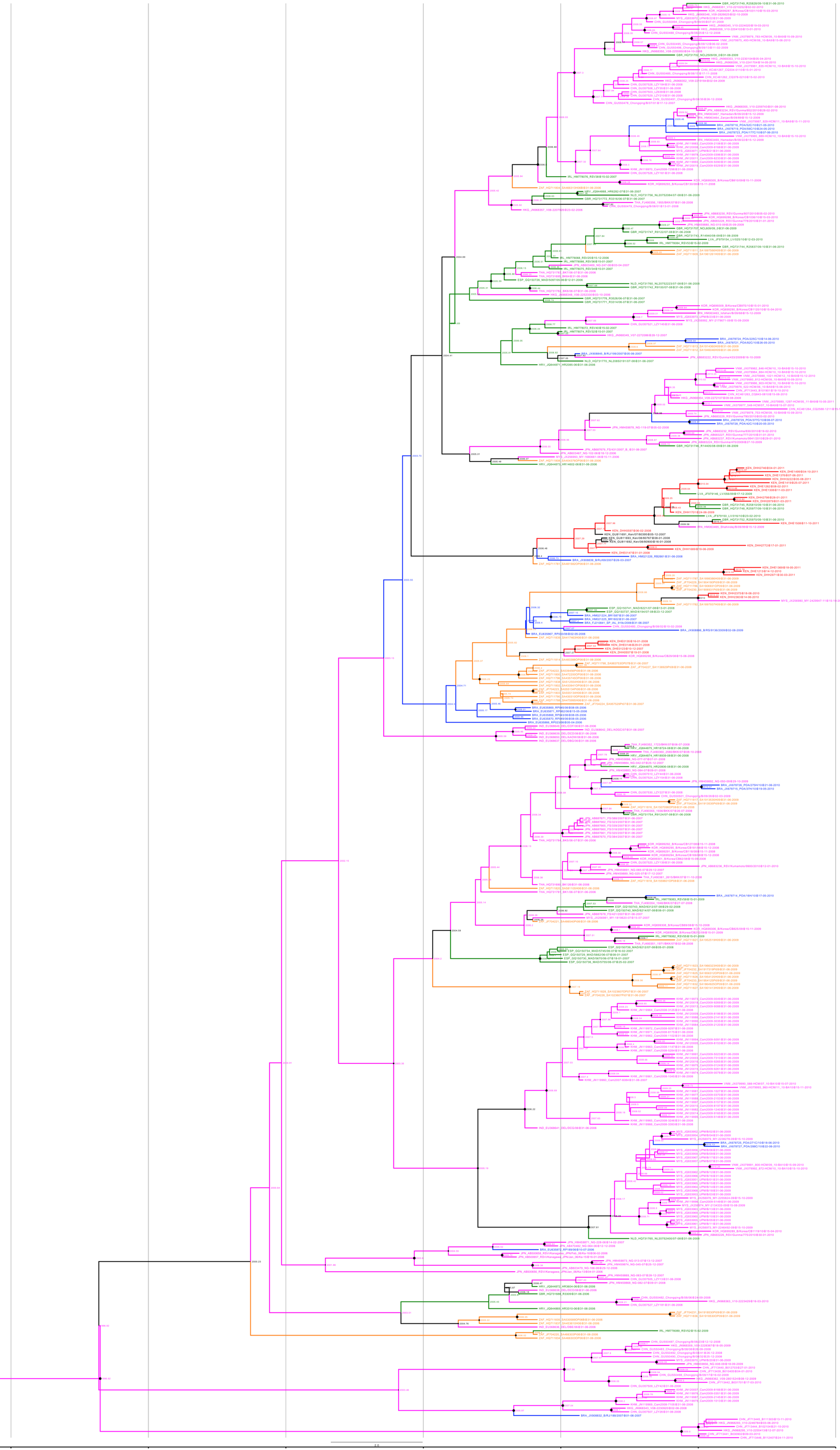

Supplement: Additional file 5: Figure A4 — A time resolved BEAST phylogenetic tree showing the relationship of the unique RSV B sequences from Dadaab and the unique RSV B sequences from 18 other countries collected from 2006-2011. The taxa are coloured by the continent the sequences were sampled. In addition to Kenya, the 18 other countries included are Brazil (BRA), China (CHI), Croatia (HRV), Great Britain and Northern Ireland (GBR), Hong Kong (HKG), India (IND), Iran (IRN), Japan (JPN), Latvia (LVA), Malaysia (MYS), Netherlands (NLD), South Africa (ZAF), Thailand (THA), Cambodia (KHM), Spain (ESP), Ireland (IRL), Vietnam (VNM), and South Korea (KOR). The Dadaab sequences are coloured in red, Kilifi in black. The taxon nomenclature includes three-letter abbreviation for the country of sampling/accession number/strain name. [file 1471-2334-14-178-S5.pdf]
